# Supplementary material for: Drug transport kinetics of intravascular triggered drug delivery systems
Source: Commun Biol. 2021 Jul 28;4:920. doi: 10.1038/s42003-021-02428-z (PMC8319190; doi:10.1038/s42003-021-02428-z)
Supplement: Supplementary file 1 — Supplementary Information [file 42003_2021_2428_MOESM1_ESM.pdf]

Supplementary Information

Drug Transport Kinetics of Intravascular Triggered Drug Delivery Systems

Timo L.M. ten Hagen[1], Matthew R. Dreher[2], Sara Zalba[1], Ann L.B. Seynhaeve[1], Mohamadreza Amin[1], Li Li[1], Dieter Haemmerich[3,4*]

Affiliations:
[1] Laboratory Experimental Oncology and Nanomedicine Innovation Center Erasmus (NICE), Department of Pathology, Erasmus Medical Center, Rotterdam, The Netherlands
[2] Boston Scientific, Marlborough, MA, USA
[3] Department of Pediatrics, Medical University of South Carolina, Charleston, SC, USA
[4] Department of Bioengineering, Clemson University, Clemson, SC, USA
* Corresponding author. Email address: haemmerich@ieee.org

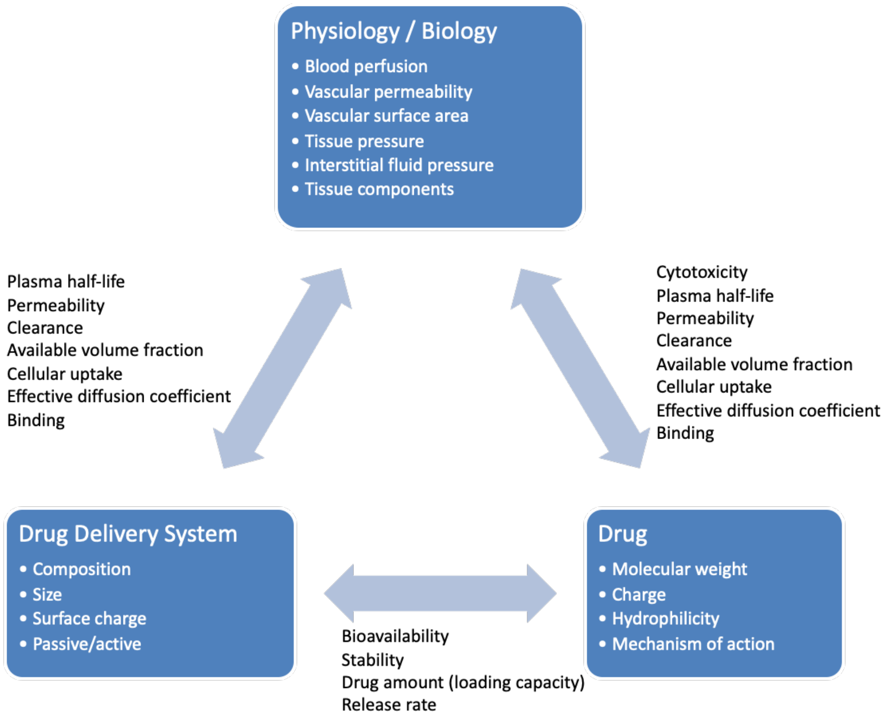

Fig. S1. Schematic depicting the interplay between three categories: physiology/biology, drug delivery system (DDS), and drug. The terms in each box are often used to describe or characterize each category. The terms adjacent to each arrow highlight the transport properties that are influenced by the interplay between two categories. For example, the plasma half-life, permeability, and clearance of a DDS depend on the interaction between the physiology/biology and the DDS. Depending on the state of the drug (i.e. DDS-associated or free), the transport properties may be different (e.g. half-life). Stimuli may be used to change the transport kinetics by changing the drug state from DDS-associated to free. Note, this is not a complete list.

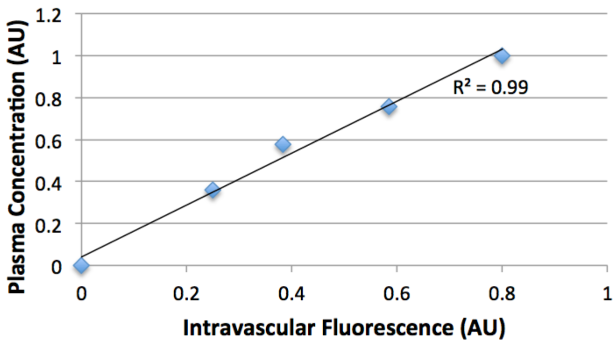

Fig. S2. *In vivo* fluorescence linearity. Intravascular fluorescence was compared to plasma concentration quantified from blood samples at various time points after administration of unencapsulated dye (data in Fig. S5).

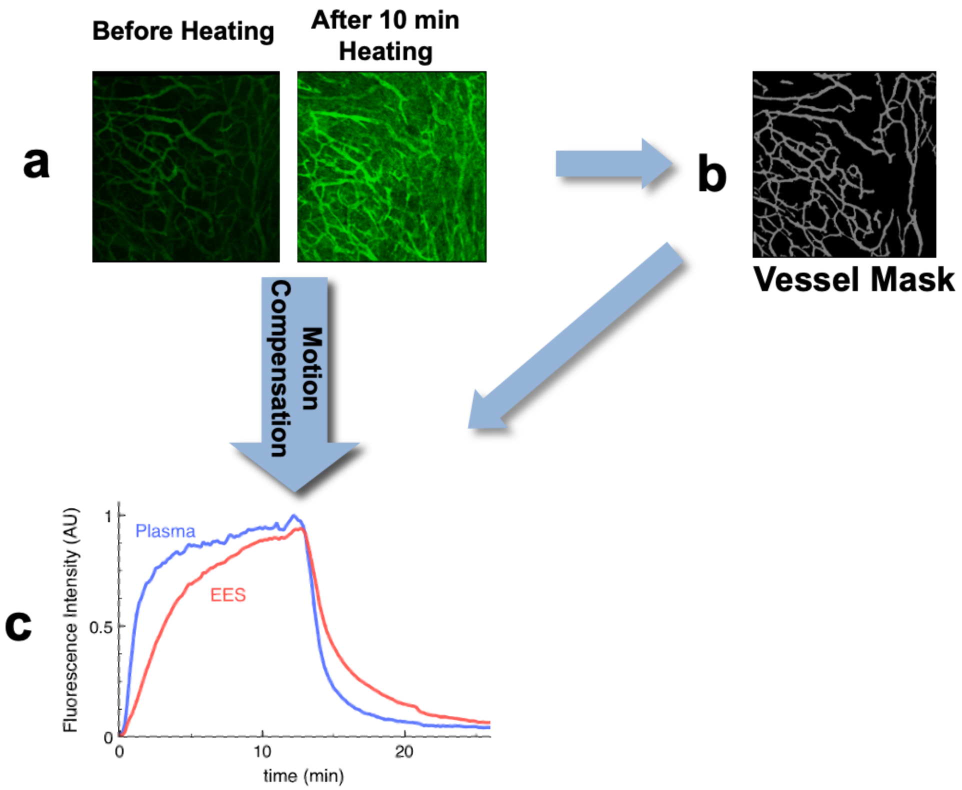

Fig. S3. Intravital image processing overview. a Fluorescence images (920x920 μm FOV) with TSL-encapsulated carboxyfluorescein (green) dye, before and after 10 min heating. **b** Segmented vasculature network. **c** Mean intra- and extravascular fluorescence intensity.

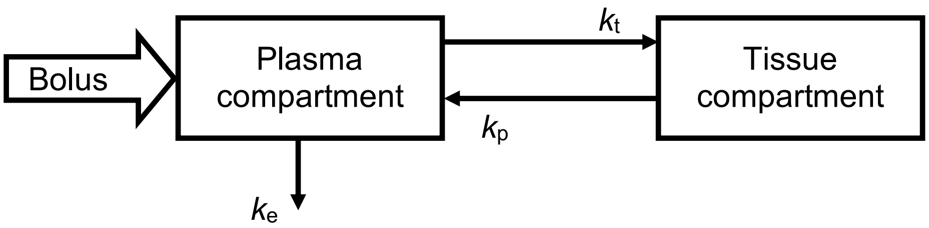

Fig. S4. Pharmacokinetic (PK) model for PK parameter calculation. A two-compartment model was used for calculation of clearance and distribution parameters for free (unencapsulated) dye.

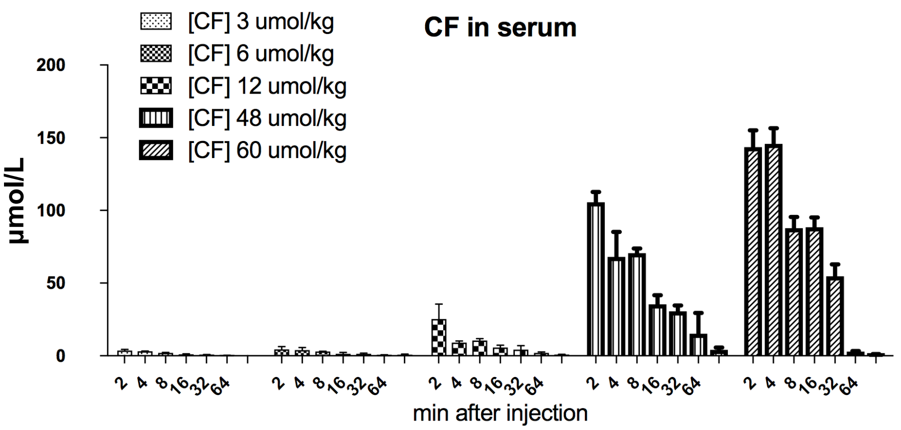

Fig. S5. Plasma clearance. Clearance of free (unencapsulated) carboxyfluorescein (CF) dyz, measured at 5 different doses. Error bars indicated standard deviation (n=3 animals at each dose/time point)

We normalized the plasma concentration data (Fig. S5) for each dose relative to the first time point at 2 min (Fig. S6). Subsequently, a bi-exponential fit (Equ. 7) was made (Fig. S7) from which the distribution and clearance rate constants were calculated (Equ. 8).

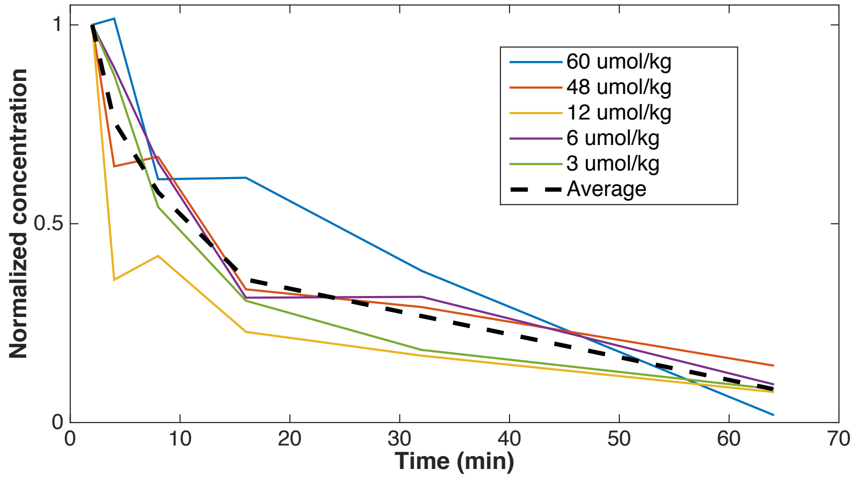

Fig. S6. Normalized clearance data. Clearance and distribution rate constants were calculated based on normalized clearance data. Shown is normalized data for 5 doses, and average based on data from Fig. S5.

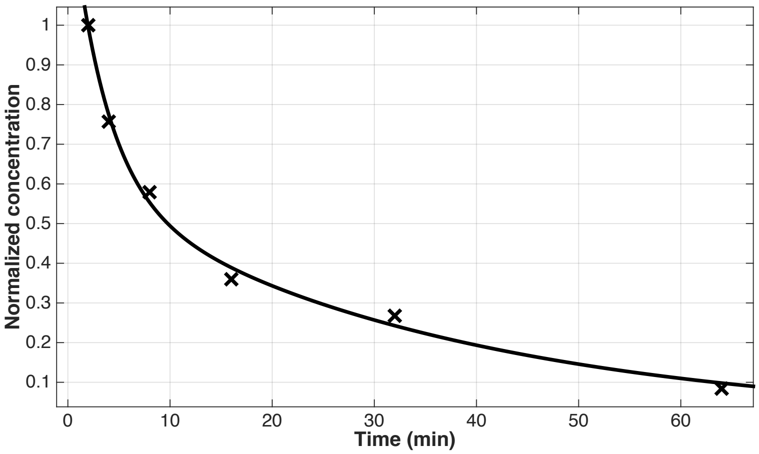

Fig. S7. Biexponential fit to clearance data. Normalized mean plasma concentration (average of 5 doses from Fig. S6) (x markers), and fitted bi-exponential curve from Equ. 7 (R^2=0.99), with following parameters: C_0=1.363; α=0.4407; λ_1=0.02832; λ_2=0.2903.

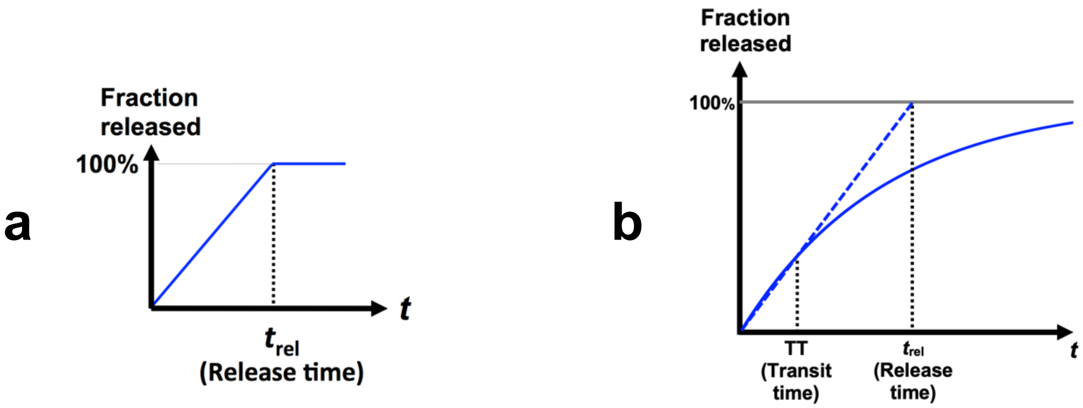

Fig. S8. IV-DDS release kinetics. a We assumed zero-order release kinetics for IV-DDS as shown on the graph, with release time (t_{rel}) indicating time required for complete release. **b** For IV-DDS with non-zero-order release kinetics, release time (t_{rel}) can be approximated if release kinetics is approximately linear up to the transit time (*TT*; in our tumor model, *TT*=5 s). The estimated release time (t_{rel}) is where the linear approximation intersects with 100% release. This approximation is adequate since release from IV-DDS only occurs during transit of the tumor region (i.e. up to the transit time (*TT*)). After that, IV-DDS return to systemic circulation without further release. We used this approximation to estimate release time (t_{rel}) for sTSL and fTSL (see Fig. S10-12; Table S3).

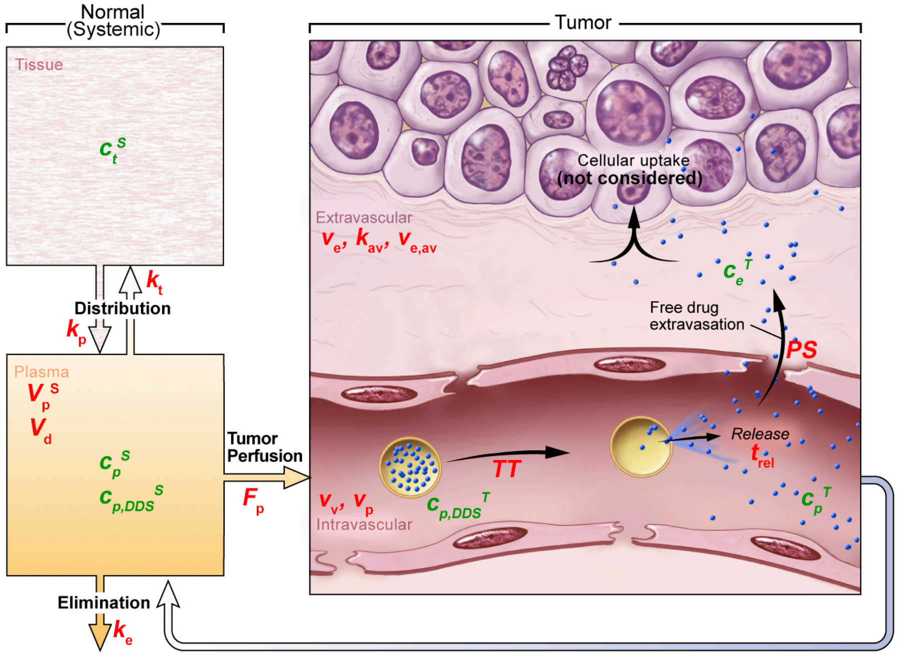

Fig. S9. Schematic of computational model. Model parameters are indicated in red, and model variables are indicated in green (see Table 3 and Table S2 for parameter and variable descriptions).

Table S1. Comparison of *in vivo* studies and computer model. Error values are calculated relative to experimental *in vivo* results for both the studies with unencapsulated (free) drug, and the studies with thermosensitive liposomes (TSL).

	**Parameter [units]**	***In vivo***	**Computer model**	**Error of computer model**	**Parameter description**
	c_{e_peak} [µg/ml]	5.4 ± 0.3	5.7	0.3 (4.0%)	Peak EES concentration
**Free drug studies**	AUC c_e [µg/ml*min]	56.9	58.2	1.3 (2.4%)	AUC of EES concentration
	c_e(t) [µg/ml]	-	-	0.2 (3.8%)*	Mean absolute error of c_e(t), t=0…20 min (Normalized error (%))
	$c_{plateau,fTSL}$ [µg/ml]	25.9 ± 5.8	18.2	7.6 (29.5%)	Plateau EES concentration
	AUC $c_{e,fTSL}$ [µg/ml*min]	192.5	135.6	56.8 (29.5%)	AUC of EES concentration
	err_{fTSL} [µg/ml]	-	-	4.0 (14.9%)*	Mean absolute error of c_e(t), t=0…20 min (Normalized error (%))
**TSL studies**	$c_{plateau,sTSL}$ [µg/ml]	2.5 ± 1.4	2.5	0.03 (1.4%)	Plateau EES concentration
	AUC $c_{e,sTSL}$ [µg/ml*min]	36.8	17.3	19.4 (52.9%)	AUC of EES concentration
	err_{sTSL} [µg/ml]	-	-	1.2 (48.4%)*	Mean absolute error of c_e(t), t=0…20 min (Normalized error (%))

* Normalized mean absolute error (%)
Table is divided into two sections indicated by the labels 'Free drug studies' and 'TSL studies' in bold.
Column headings are indicated by bold font.

Table S2. Parameters used in computational model.

Parameter [Units]	Value	Parameter description	Parameter source
Hct^S [1]	0.45	Systemic Hematocrit	[1]
Hct^{MV} [1]	0.19	Hematocrit inside tumor microvasculature	[2]
v_v [1]	0.29	Tumor blood volume fraction (imaging window)	Intravital Imaging
v_p^T [1]	0.16	Tumor plasma volume fraction (whole tumor)	$v_p^T=v_v*(1-HcT^S)$
v_p [1]	0.23	Tumor plasma volume fraction (imaging window)	$v_p^T=v_v*(1-HcT^{MV})$
v_e [1]	0.71	Tumor extravascular volume fraction (imaging window)	Intravital Imaging
k_{av} [1]	0.39	Available volume fraction in extravascular space (for unencapsulated dye)	Intravital Imaging
$v_{e,av}$ [1]	0.28	Available extravascular tumor volume fraction (28% of extravascular volume is available to dye for distribution)	$v_{e,av} = k_{av} * v_e$
t_{rel} [s]	8.2 (fTSL) 63.0 (sTSL)	IV-DDS release time at 42ºC (time to complete release assuming zero-order kinetics)	*In vitro* measurement
TT [s]	5.0	Tissue transit time of tumor segment	Intravital Imaging
V_p^S [µL]	1120	Systemic plasma volume (=IV-DDS volume of distribution)	Calculated based on animal weight (20 g) [3]
VT [µL]	10	Tumor volume	Measured in animals
V_p^T [µL]	0.16	Tumor plasma volume	$V_p^T = VT * v_p^T$
ID [µg]	60	Injected dose of IV-DDS encapsulated drug (based on 20 g animal weight, and a dose of 8 µmol/kg)	defined
PS [1/s]	0.012	Vascular permeability-surface area product (*P* represents the apparent permeability rather than true vascular permeability)	Intravital Imaging
F_p [mL/(mL*s)]	0.047	Tumor plasma perfusion rate (imaging window)	Intravital Imaging
V_D [mL]	8.99	Initial volume of distribution for unencapsulated drug (Peak plasma concentration=(Injected Dose)/V_D)	Serial blood sampling
k_p [s^{-1}]	$1.29 * 10^{-3}$	Transport rate constant, tissue -> plasma	Serial blood sampling
k_t [s^{-1}]	$0.90 * 10^{-3}$	Transport rate constant, tissue -> plasma	Serial blood sampling
k_e [s^{-1}]	$0.80 * 10^{-3}$	Elimination rate constant	Serial blood sampling

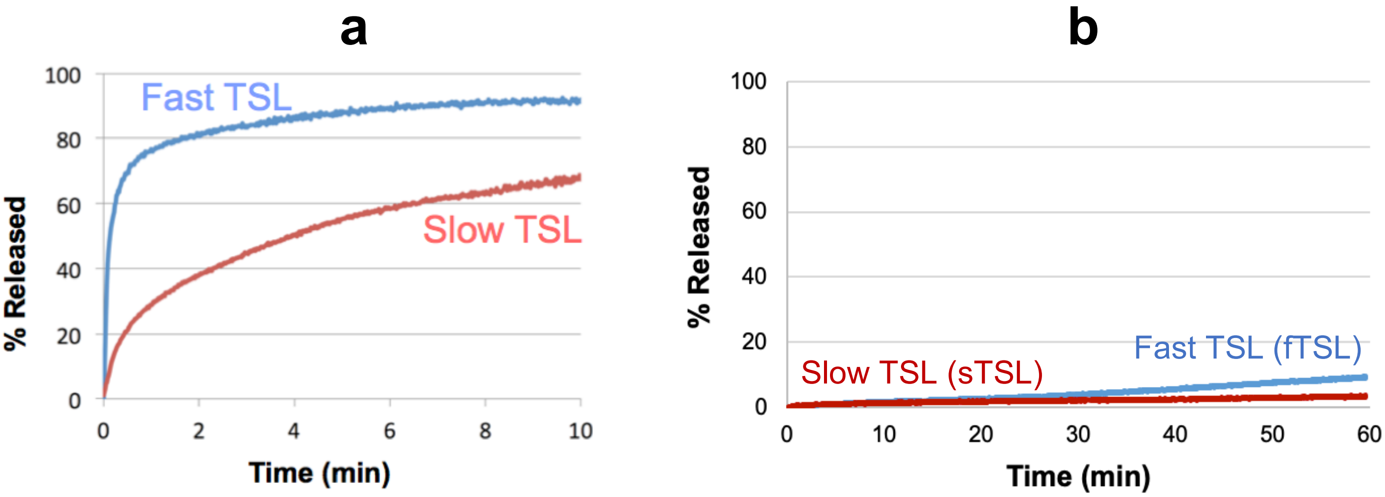

Fig. S10. TSL release kinetics and stability in fetal calve serum (FCS). a Release kinetics of fast (fTSL) and slow releasing TSL (sTSL) at 42ºC, over 10 min. **b** Encapsulation stability of fTSL and sTSL at 37ºC, over 60 min.

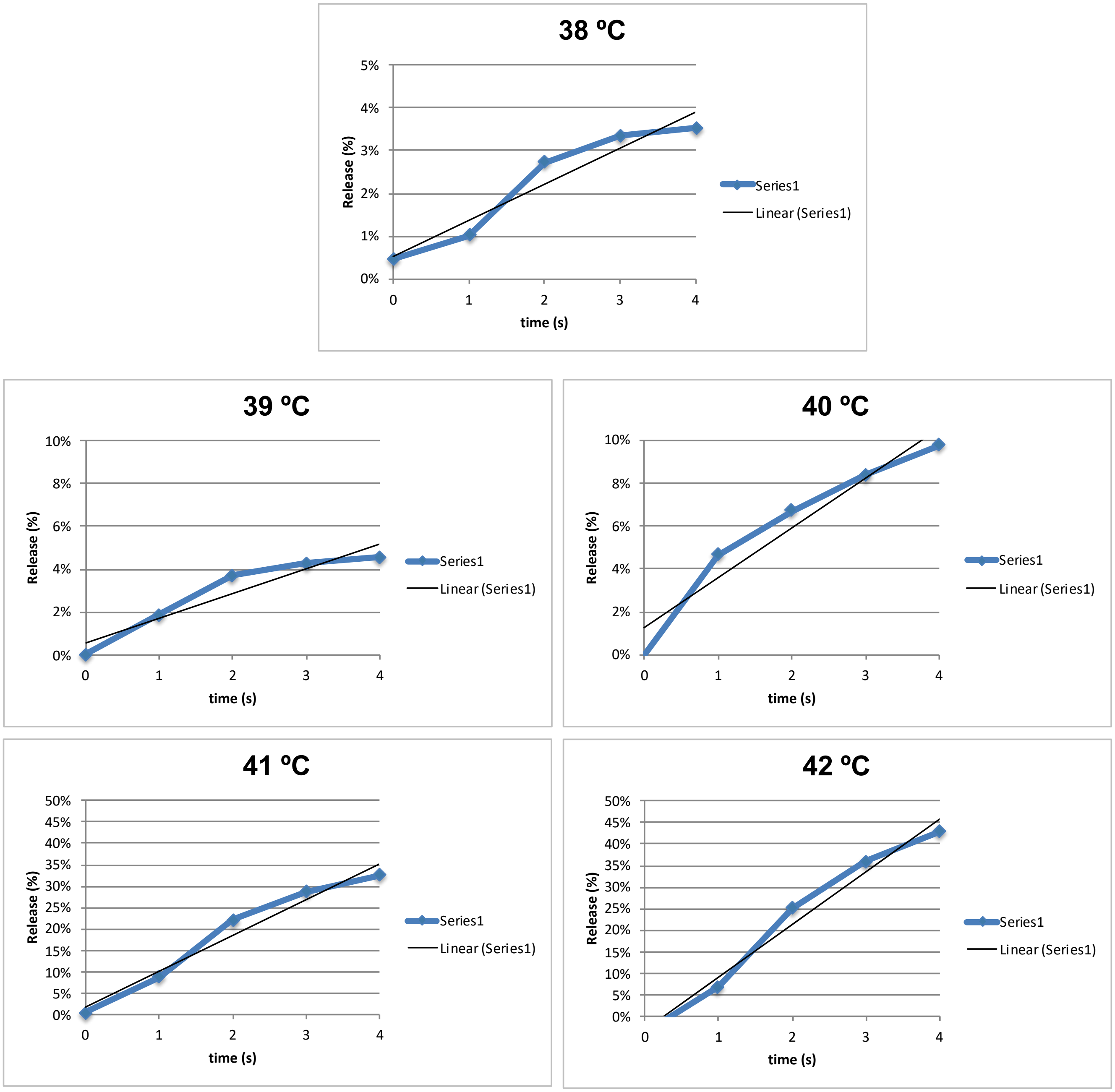

Fig. S11. fTSL release kinetics. Shown during first 4 seconds for temperatures of 38 to 42ºC. Linear approximation is indicated as black line, and release time was calculated based on the slope of this linear approximation (t_{rel}=1/R, where *R* is the slope of the linear approximation).

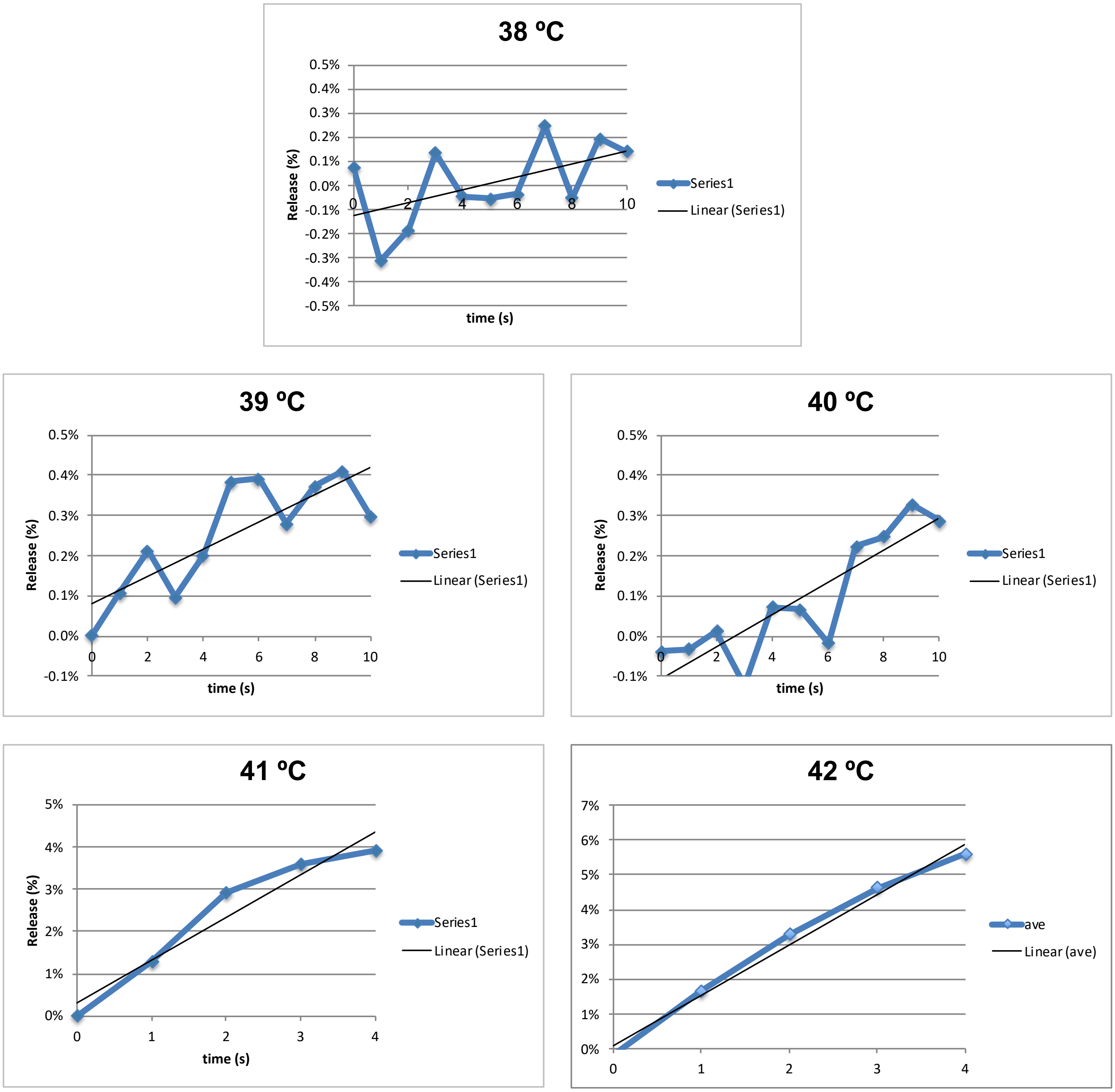

Fig. S12. sTSL release kinetics. Shown during first 10 seconds for temperatures of 38 to 40ºC, and for first 4 seconds for 41 and 42ºC. Linear approximation is indicated as black line, and release time was calculated based on the slope of this linear approximation (t_{rel}=1/R, where R is the slope of the linear approximation).

Table S3. Release time in sec for fTSL and sTSL for temperatures from 38 to 42ºC.

Temperature [ºC]	fTSL release time (t_{rel}) [s]	sTSL release time (t_{rel}) [s]
38	104.9	2857.1
39	86.7	2409.6
40	43.9	466.2
41	11.9	96.1
42	8.2	63.0

Supplementary Note 1

Global sensitivity analysis

We performed global sensitivity analyses based on the computer model parameter variability indicated in Table S4, both for unencapsulated drug and for thermosensitive liposomes (fTSL). We calculated (1) main effects, which indicate the contribution of an individual parameter on output variance, and (2) total effects, which indicate both the individual contribution of a parameter and it's interactions with other parameters on output variance. As output we considered the root-mean-square error (RMSE) of the tumor interstitial (EES) concentration calculated over 20 min, in comparison to the reference EES concentration based on the parameter means (Equ. 19).

The results for unencapsulated drug suggest that the variability in tumor drug uptake is primarily dependent on pharmacokinetic parameter variation, i.e. mainly affected by systemic distribution and elimination (Fig. S13). In contrast, for TSL the pharmacokinetic parameters have little impact. Tumor drug uptake variability for TSL (i.e. IV-DDS) is primarily dependent on the variability of tumor transport parameters (transit time (*TT*), permeability-surface area product (*PS*)), and on TSL release time (t_{rel}))(Fig. S14). These three parameters are also present in the two indices (release index (*R.I.*), permeability index (*P.I.*)) that have been identified as dictating tumor drug uptake, providing additional evidence for the relevance of these indices.

Table S4 Parameters and parameter variability used in uncertainty and sensitivity analyses. The mean value and variability (standard deviation) are indicated for each parameter based on our *in vivo* data.

Parameter [Units]	Value	Parameter description	Parameter source
v_p [1]	0.23 ± 0.07	Tumor plasma volume fraction (imaging window)	Intravital Imaging
v_e [1]**	0.71 ± 0.09	Tumor extravascular volume fraction (imaging window)	Intravital Imaging
k_{av} [1]**	0.39 ± 0.13	Available volume fraction in extravascular space (for unencapsulated dye)	Intravital Imaging
$v_{e,av}$ [1]	0.28 ± 0.13	Available extravascular tumor volume fraction (28% of extravascular volume is available to dye for distribution)	$v_{e,av} = k_{av} * v_e$
v_p^T [1]	0.16 ± 0.05	Tumor plasma volume fraction (whole tumor)	Calculated (see Table S2)
t_{rel} [s]*	8.2 ± 0.8	IV-DDS release time at 42ºC (fTSL) (time to complete release assuming zero-order kinetics)	*In vitro* measurement
TT [s]	5.0 ± 0.5	Tissue transit time of tumor segment	Intravital Imaging
V_p^S [µL]	1120 ± 16	Systemic plasma volume (=IV-DDS volume of distribution)	Calculated based on animal weight (20 g) [3]
VT [µL]*	10 ± 1	Tumor volume	Measured in animals
V_p^T [µL]**	0.30 ± 0.02	Tumor plasma volume	$V_p^T = VT * v_p^T$
PS [1/s]	0.012 ± 0.005	Vascular permeability-surface area product (*P* represents the apparent permeability rather than true vascular permeability)	Intravital Imaging
F_p [mL/(mL*s)]**	0.047 ± 0.02	Tumor plasma perfusion rate (imaging window)	$F_p = v_p^T/TT$
V_D [mL]	8.99 ± 0.58	Initial volume of distribution for unencapsulated drug (Peak plasma concentration=(Injected Dose)/V_d)	Serial blood sampling
k_p [s^{-1}]	1.29 ± 0.52 *10^{-3}	Transport rate constant, tissue -> plasma	Serial blood sampling
k_t [s^{-1}]	0.90 ± 0.22 *10^{-3}	Transport rate constant, tissue -> plasma	Serial blood sampling
k_e [s^{-1}]	0.80 ± 0.30 *10^{-3}	Elimination rate constant	Serial blood sampling

* For the parameters indicated, no experimental data was available to estimate variability and we assumed a standard deviation equal to 10% of the mean.

** These parameters can be derived from other parameters in this table, and were therefore not explicitly included in the sensitivity analysis.

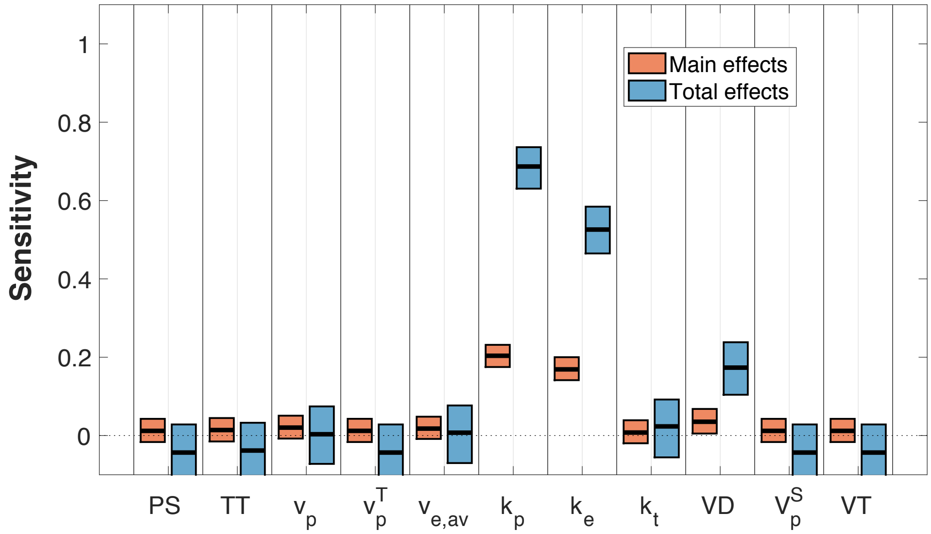

Fig. S13. Global sensitivity analysis results for computational model of unencapsulated drug. Parameters are listed at the bottom. "Main Effect" indicates the first-order effect of a parameter, and "Total Effect" includes the first-order effect as well as interaction effects with any other parameter(s) (of any order). Colored bars indicate bounds for each effect. The output variability is primarily dictated by the pharmacokinetic parameters describing distribution and elimination of free drug (k_p, k_e, V_D).

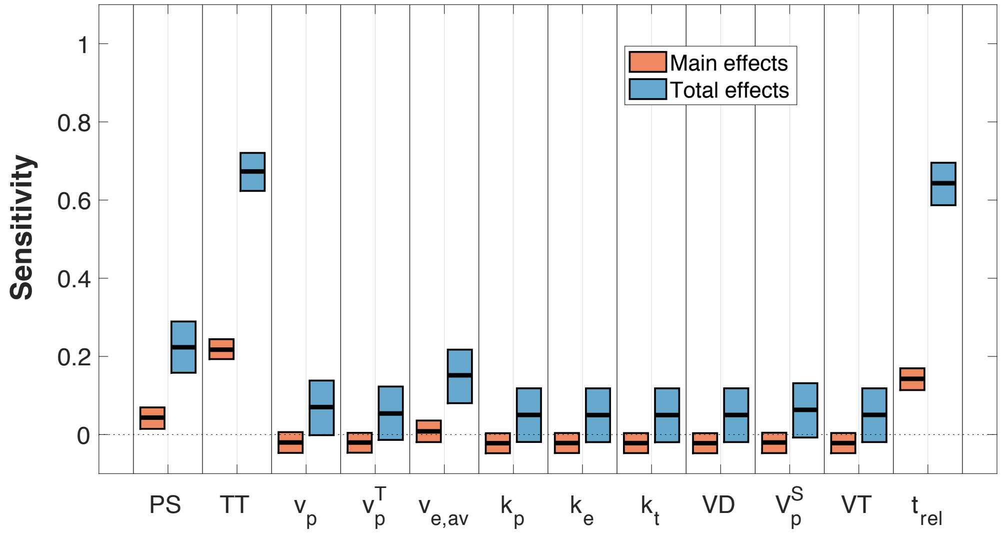

Fig. S14. Global sensitivity analysis results for computational model of IV-DDS encapsulated drug (fTSL). Parameters are listed at the bottom. "Main Effect" indicates the first-order effect of a parameter, and "Total Effect" includes the first-order effect as well as interaction effects with any other parameter(s) (of any order). Colored bars indicate bounds for each effect. The output variability is primarily dictated by two tumor transport parameters (permeability-surface area product (*PS*), tumor transit time (*TT*)), and by IV-DDS release time (t_{rel}).

Table S5. Hepatic first-pass extraction fraction (*EF*) for common chemotherapy agents [4-8]. The ratio PS/F_p was calculated from *EF* based on Equ. 1. *EF* in tumors may differ from the hepatic *EF* indicated here, but is usually not available due to the difficulty in measuring tumor *EF*.

Agent	Hepatic Extraction Fraction (EF)	Ratio PS/F_p
FUDR	0.94-0.99	2.8-4.6
Paclitaxel	0.95	3.0
Gemcitabine	0.55-0.89	0.8-2.2
Doxorubicin	0.4-0.5	0.5-0.7
Oxiplatin	0.5	0.6
5-FU	0.3-0.4	0.4-0.5
Cisplatin	0.24-0.27	0.3
Mitomycin C	0.1-0.2	0.1-0.2

Table S6. Characterization of thermosensitive liposomes. Both sTSL and fTSL were analyzed by dynamic light scattering (DLS).

Liposome formulation	Size (nm)	Polydispersity index (PDI)	Zeta potential (mV)	Loading efficiency (%)
sTSL	83.0 ± 1.1	0.054 ± 0.017	-11.30 ± 1.79	3.37 ± 0.44
fTSL	82.8 ± 3.6	0.045 ± 0.016	-12.56 ± 2.11	3.18 ± 0.37

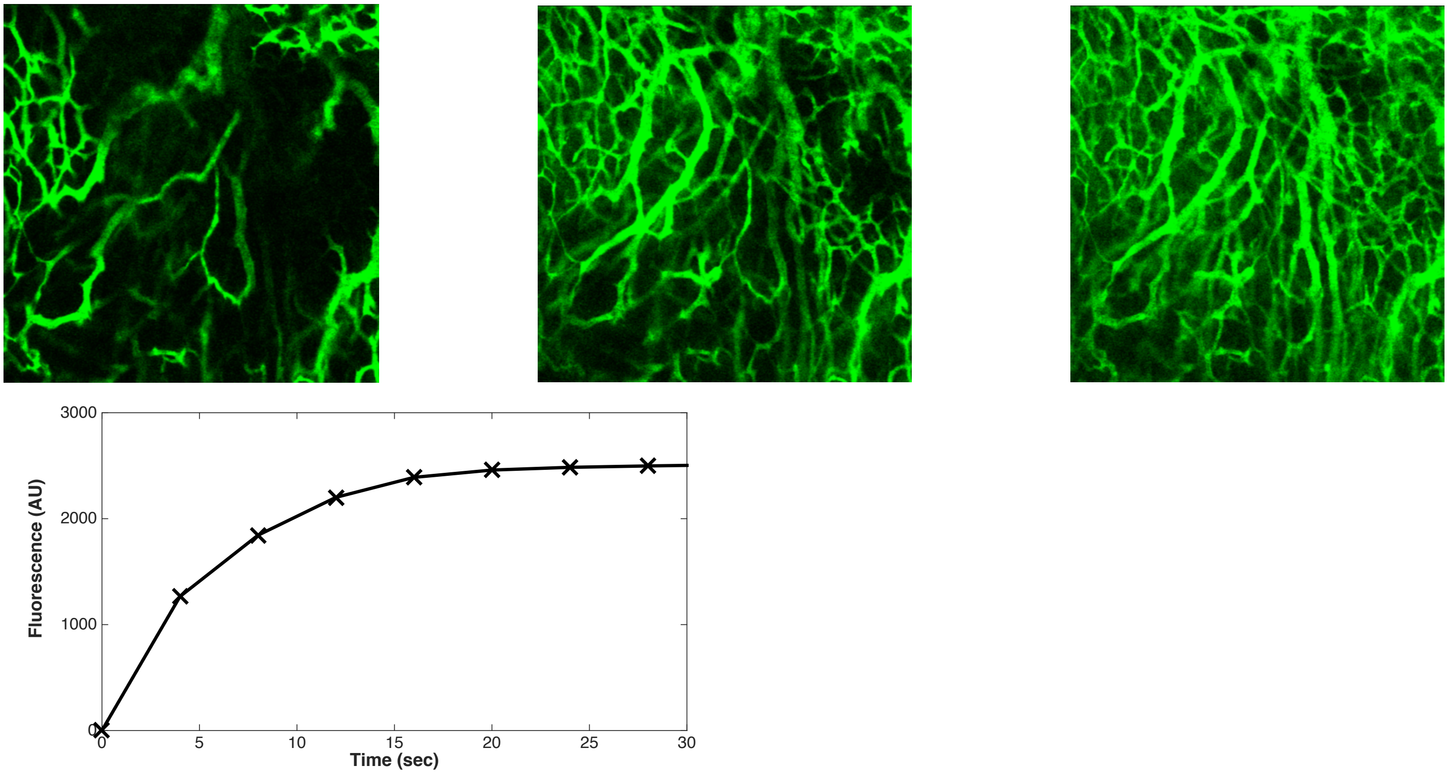

Fig. S15. Tissue transit time calculation example. Upper panels show intravital images (921 x 921 µm) following bolus administration of free carboxyfluorescein (CF) dye after 4 s, 12 s, and 20 s. Lower graph shows the corresponding intravascular signal $I_v(t)$ during the first 30 s. The transit time (*TT*) of this tissue segment was calculated by deconvolution of $I_v(t)$ as described in the methods, and *TT*=4.6 s for this dataset.

Supplementary Note 2

Computer model considering varying release times due to recirculation of IV-DDS

We created an extended computer model where we implemented the consideration of multiple IV-DDS populations, depending on whether IV-DDS had already passed through the tumor. However, this extended model is only applicable for the simplified case of IV-DDS having a constant release time (e.g. assuming a constant temperature throughout triggered release if thermosensitive liposomes (TSL) are used). The issue of multiple passes is relevant for IV-DDS that do not conform perfectly to 0th order release kinetics under certain conditions, such as the fTSL and sTSL employed here (see Fig. S10). In such a case, if an IV-DDS passes the tumor a second time, the release time will be different from the first time. We assumed the specific release kinetics of our example IV-DDS (fTSL and sTSL), and as mentioned assumed a simplified case where release times and temperature are constant during the release duration. This assumption considerably simplifies the model, since then each IV-DDS population that passed through the tumor a certain number of times has the same release time assigned – i.e. resulting in a very limited number of IV-DDS populations that need to be considered, rather than an infinite number of populations as would be required for a generalized model. This approach takes advantage of the fact that all IV-DDS that passed through the tumor once release the same amount of drug; the same is true for IV-DDS passing through the tumor a second and third time. IV-DDS with more than three passes were ignored, as they represent <<0.01% of all IV-DDS based on our model results. Each of these three IV-DDS populations was assigned a specific release time based on our experimental data, where we measured release time of fast releasing (fTSL) and slow releasing thermosensitive liposomes (sTSL) at 42ºC. We determined the release time as described above, but now measured three release times, for liposomes that had passed through the tumor zero, once, or twice, respectively. Accordingly, we made a linear fit to the TSL release data (1) during the first 5 s (=tumor transit time, i.e. the time a liposomes would spend in the tumor during a single pass), (2) from 5-10 s, (3) and from 10-15 s (Table S7).

Table S7. Release time for fTSL and sTSL at 42ºC, with 0, 1 and 2 prior passes through the tumor.

Prior tumor passes	fTSL release time (t_{rel}) [s]	sTSL release time (t_{rel}) [s]
0	8.2	63.0
1	38.0	47.5
2	94.3	43.5

We added the following equations, where we represent IV-DDS populations individually that passed the tumor already zero, one, or two times (as mentioned, IV-DDS entering with >2 prior passes represent <<0.01% of all IV-DDS, and were ignored):

$$\frac{dc^S_{p\_DDS0}(t)}{dt} = -\frac{V^T_p}{V^S_p}\frac{F_p}{v_p} c^S_{p\_DDS0}(t) \quad \text{(Equ. S1)}$$

$$\frac{dc^S_{p\_DDS1}(t)}{dt} = \frac{V^T_p}{V^S_p}\frac{F_p}{v_p}\left(c^S_{p\_DDS0}(t) - c^S_{p\_DDS1}(t)\right) \quad \text{(Equ. S2)}$$

$$\frac{dc^S_{p\_DDS2}(t)}{dt} = \frac{V^T_p}{V^S_p}\frac{F_p}{v_p}\left(c^S_{p\_DDS1}(t) - c^S_{p\_DDS2}(t)\right) \quad \text{(Equ. S3)}$$

As mentioned, for IV-DDS release, each IV-DDS population was considered with it's specific release time, and Equ. 9 and Equ. 10 were modified accordingly to consider all three IV-DDS populations from Equ. S1-S3. We performed a simulation where we assumed that the tumor was instantaneously heated to 42ºC, and remained at 42ºC for 6.6 min. This scenario represents a worst case relative to our experimental conditions, where temperature slowly approached 42ºC (where release is fastest), and remained between 41-42ºC for only 6.6 min (Fig. 4a). Note that below 41ºC, release is very slow (Fig. S11), and no significant tumor drug accumulation occurs until tumor temperature reaches 41ºC (Fig. 4a,c).

Figure S15 shows the results for the standard model compared to the model considering multiple passes. The reduced release from fTSL that pass through the tumor a 2nd or 3rd time manifests as lower concentrations in tumor plasma (c_p^T) and tumor interstitium (c_e^T) (Fig. S15b). The error due to neglecting multiple passes was however small, with 2.7% mean error for fTSL, and with 0.9% mean error for sTSL (Table S8).

The volume ratio of tumor plasma (where drug release from IV-DDS occurs) to systemic plasma (which serves as reservoir of IV-DDS-encapsulated drug) in the model was ~700:1, which is based off a mouse tumor with a volume of 10 μL. For comparison, to achieve this same ratio in a human patient would require a human tumor of >6 cm diameter. I.e. the consideration of the varying release time due to recirculation of IV-DDS can be neglected not only here, but also for most human tumors since the plasma volume where drug release from IV-DDS is triggered is very small compared to the systemic plasma volume.

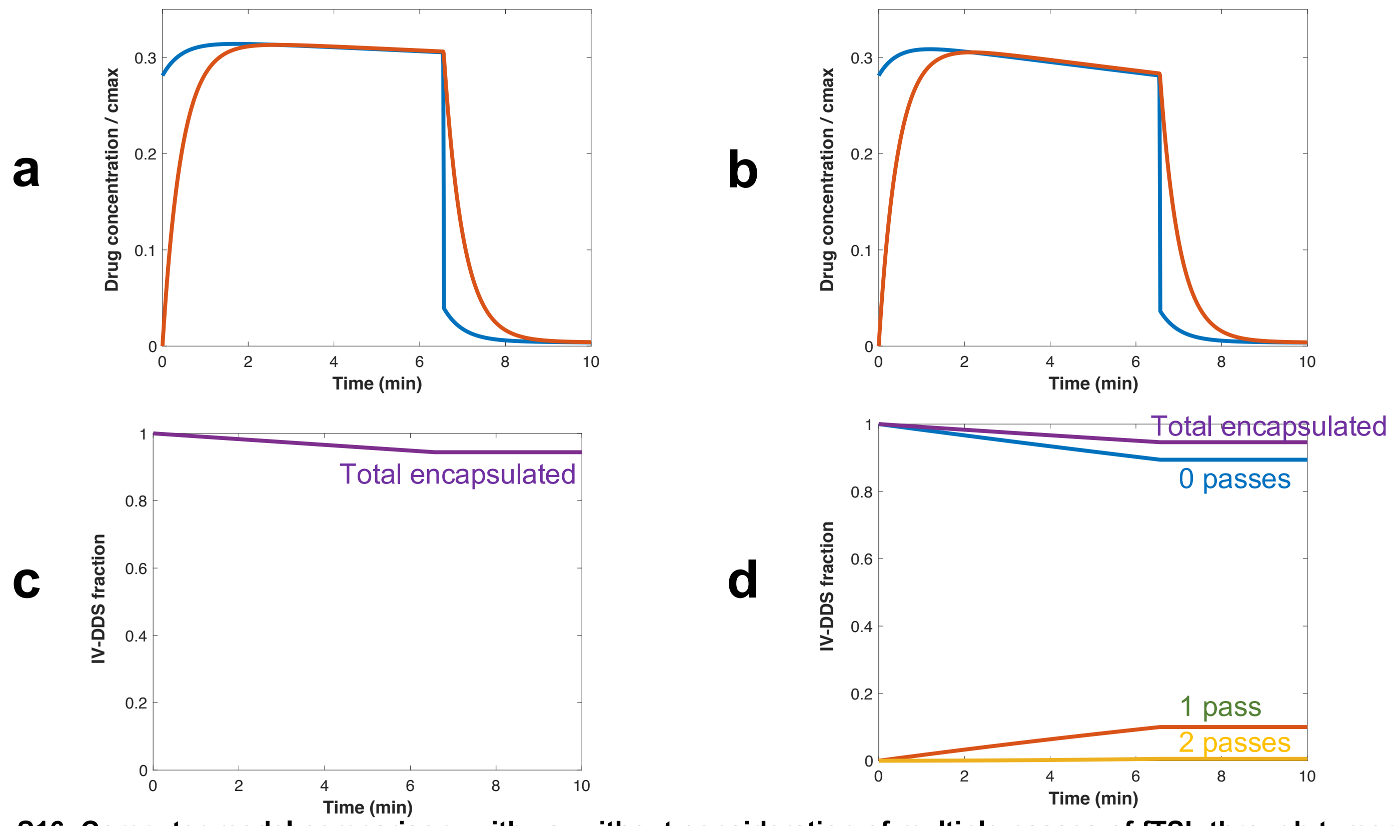

Fig. S16. Computer model comparison: with vs. without consideration of multiple passes of fTSL through tumor. fTSL were simulated with triggered release duration of 6.6 min. **a** Drug concentrations based on standard model as used in the manuscript, and **b** based on the revised model considering multiple passes of fTSL. **c** Fraction of encapsulated drug based on standard model. **d** Total fTSL-encapsulated drug, and separately indicated fTSL populations with 0, 1, and 2 prior passes through the tumor, based on model considering multiple passes of fTSL. After 10 min, 94.6% of total encapsulated drug remained in systemic circulation, with 10.0% of fTSL having passed the tumor once, and 0.6% of fTSL having passed the tumor twice. **a** and **b** show tumor plasma concentration (c_p^T) and tumor interstitial concentration (c_e^T). All concentrations in **a** and **b** are plotted relative to the maximum possible concentration (c_{max}).

Table S8. Errors due to ignoring multiple passes of IV-DDS, shown for fTSL and sTSL. Errors are based on comparing the default model used in the main text to the model considering multiple passes of IV-DDS through the tumor.

	**fTSL**	**sTSL**
error c_e^T plateau [µg/ml]	0.5 (2.5%)	0.05 (1.9%)
mean error c_e^T [µg/ml]	0.5 (2.7%)	0.02 (0.9%)
error AUC(c_e^T) [µg/ml*min]	5.1 (4.1%)	0.2 (1.4%)

Supplementary References

1 Horikoshi, S. *et al.* Water deprivation stimulates transforming growth factor-beta 2 accumulation in the juxtaglomerular apparatus of mouse kidney. *The Journal of Clinical Investigation* **88**, 2117-2122, doi:10.1172/JCI115541 (1991).

2 Brizel, D. M. *et al.* A comparison of tumor and normal tissue microvascular hematocrits and red cell fluxes in a rat window chamber model. *Int J Radiat Oncol Biol Phys* **25**, 269-276 (1993).

3 Riches, A. C., Sharp, J. G., Thomas, D. B. & Smith, S. V. Blood volume determination in the mouse. *J Physiol* **228**, 279-284 (1973).

4 de Takats, P. G., Kerr, D. J., Poole, C. J., Warren, H. W. & McArdle, C. S. Hepatic arterial chemotherapy for metastatic colorectal carcinoma. *Br J Cancer* **69**, 372-378 (1994).

5 Tsimberidou, A. M. *et al.* Phase I clinical trial of hepatic arterial infusion of paclitaxel in patients with advanced cancer and dominant liver involvement. *Cancer Chemother Pharmacol* **68**, 247-253, doi:10.1007/s00280-010-1482-y (2011).

6 Tsimberidou, A. M. *et al.* A phase 1 study of hepatic arterial infusion of oxaliplatin in combination with systemic 5-fluorouracil, leucovorin, and bevacizumab in patients with advanced solid tumors metastatic to the liver. *Cancer* **116**, 4086-4094, doi:10.1002/cncr.25277 (2010).

7 van Riel, J. M. *et al.* A phase I and pharmacokinetic study of gemcitabine given by 24-h hepatic arterial infusion. *Eur J Cancer* **45**, 2519-2527 (2009).

8 Campbell, T. N., Howell, S. B., Pfeifle, C. E., Wung, W. E. & Bookstein, J. Clinical pharmacokinetics of intraarterial cisplatin in humans. *J Clin Oncol* **1**, 755-762 (1983).
