# Supplementary material for: Drug transport kinetics of intravascular triggered drug delivery systems
Source: Commun Biol. 2021 Jul 28;4:920. doi: 10.1038/s42003-021-02428-z (PMC8319190; doi:10.1038/s42003-021-02428-z)
Supplement: Supplementary file 5 — Reporting Summary [file 42003_2021_2428_MOESM5_ESM.pdf]

nature research

Corresponding author(s): Dieter Haemmerich

Last updated by author(s): May 20, 2021

Reporting Summary

Nature Research wishes to improve the reproducibility of the work that we publish. This form provides structure for consistency and transparency in reporting. For further information on Nature Research policies, see our Editorial Policies and the Editorial Policy Checklist.

Statistics

For all statistical analyses, confirm that the following items are present in the figure legend, table legend, main text, or Methods section.

n/a	Confirmed	
☐	☒	The exact sample size (*n*) for each experimental group/condition, given as a discrete number and unit of measurement
☐	☒	A statement on whether measurements were taken from distinct samples or whether the same sample was measured repeatedly
☒	☐	The statistical test(s) used AND whether they are one- or two-sided<br>*Only common tests should be described solely by name; describe more complex techniques in the Methods section.*
☒	☐	A description of all covariates tested
☒	☐	A description of any assumptions or corrections, such as tests of normality and adjustment for multiple comparisons
☐	☒	A full description of the statistical parameters including central tendency (e.g. means) or other basic estimates (e.g. regression coefficient) AND variation (e.g. standard deviation) or associated estimates of uncertainty (e.g. confidence intervals)
☒	☐	For null hypothesis testing, the test statistic (e.g. *F*, *t*, *r*) with confidence intervals, effect sizes, degrees of freedom and *P* value noted<br>*Give P values as exact values whenever suitable.*
☒	☐	For Bayesian analysis, information on the choice of priors and Markov chain Monte Carlo settings
☒	☐	For hierarchical and complex designs, identification of the appropriate level for tests and full reporting of outcomes
☒	☐	Estimates of effect sizes (e.g. Cohen's *d*, Pearson's *r*), indicating how they were calculated

Our web collection on statistics for biologists contains articles on many of the points above.

Software and code

Policy information about availability of computer code

Data collection	Zeiss LSM
Data analysis	MeVisLab v2.7.3; Matlab vR2020a; Microsoft Excel Mac v16.43

For manuscripts utilizing custom algorithms or software that are central to the research but not yet described in published literature, software must be made available to editors and reviewers. We strongly encourage code deposition in a community repository (e.g. GitHub). See the Nature Research guidelines for submitting code & software for further information.

Data

Policy information about availability of data

All manuscripts must include a data availability statement. This statement should provide the following information, where applicable:

- Accession codes, unique identifiers, or web links for publicly available datasets
- A list of figures that have associated raw data
- A description of any restrictions on data availability

Data not included in main text or supplementary materials are available from authors upon reasonable request.

Field-specific reporting

Life sciences study design

All studies must disclose on these points even when the disclosure is negative.

Sample size	All animal studies were replicated in separate animals (n=3), which provides a 90% confidence interval of +/-0.95 standard deviations. Since the purpose of the in vivo studies was validation of the computer models, no statistical comparisons between individual groups in animal studies were performed.
Data exclusions	No data exclusion conducted
Replication	Same experiment was done repeatedly (at least three times)
Randomization	Mice were pooled and selected randomly; produced liposomal nanoformulations were used randomly (after QC)
Blinding	Blinding not done at EMC site (site of data collection). Data was analyzed at MUSC with knowledge of experimental conditions.

Reporting for specific materials, systems and methods

We require information from authors about some types of materials, experimental systems and methods used in many studies. Here, indicate whether each material, system or method listed is relevant to your study. If you are not sure if a list item applies to your research, read the appropriate section before selecting a response.

Materials & experimental systems

n/a	Involved in the study	
☒	☐	Antibodies
☐	☒	Eukaryotic cell lines
☒	☐	Palaeontology and archaeology
☐	☒	Animals and other organisms
☒	☐	Human research participants
☒	☐	Clinical data
☒	☐	Dual use research of concern

Methods

n/a	Involved in the study	
☒	☐	ChIP-seq
☒	☐	Flow cytometry
☒	☐	MRI-based neuroimaging

Eukaryotic cell lines

Policy information about cell lines

Cell line source(s)	Provided by colleague
Authentication	No authentication was performed. Tumors were grown from tumor pieces extracted from donor mice, and authentication in these tissue samples was not possible.
Mycoplasma contamination	Cell were tested negatively
Commonly misidentified lines (See ICLAC register)	N/A

Animals and other organisms

Policy information about studies involving animals; ARRIVE guidelines recommended for reporting animal research

Laboratory animals	Mice, C57bl/6, male, 12 weeks or older
Wild animals	No wild animals
Field-collected samples	No field-collected samples
Ethics oversight	Experiments involving mice and genetically modified organisms are performed according to all relevant governmental and institutional guidelines and regulations, in compliance with protocols approved by local animal ethics committees. Animal experiments, presented in this report, were approved by the Instantie voor Dierenwelzijn Erasmus MC and conducted with permissions granted by the Nederlandse Dierexperimentencommissie (approval Nr AVD101002016792). The experiments were performed according to the European directive 2010/63/eu on the protection of animals used for scientific purposes.

Note that full information on the approval of the study protocol must also be provided in the manuscript.
