# Supplementary material for: Drug transport kinetics of intravascular triggered drug delivery systems
Source: Commun Biol. 2021 Jul 28;4:920. doi: 10.1038/s42003-021-02428-z (PMC8319190; doi:10.1038/s42003-021-02428-z)
Supplement: Supplementary file 7 — Description of Supplementary Files [file 42003_2021_2428_MOESM7_ESM.pdf]

Description of Additional Supplementary Files

File name: Suppl. Movie 1.

Description: Intravital image dataset recorded after bolus administration of unencapsulated dye. Images were acquired every 4 s, and movie runs at 3 frames/s. Movie shows the first 2 min of the 20 min dataset. Movie corresponds to data shown in Fig. 2a, b, c, e.

File name: Suppl. Movie 2.

Description: Intravital image dataset recorded after administration of fast-release thermosensitive liposomes (fTSL) before, during and after hyperthermia (20 min total). Images were acquired every 10 s, and movie runs at 6 frames/s. Movie corresponds to data shown in Fig. 4b, c, e.

File name: Suppl. Movie 3.

Description: Intravital image dataset recorded after administration of slow-release thermosensitive liposomes (sTSL) before, during and after hyperthermia (20 min total). Images were acquired every 10 s, and movie runs at 6 frames/s. Movie corresponds to data shown in Fig. 4b, c, e.
